# The Role of Neoadjuvant Immunotherapy in the Management of Merkel Cell Carcinoma with Clinically Detected Regional Lymph Node Metastasis

**DOI:** 10.1245/s10434-024-15478-4

**Published:** 2024-06-01

**Authors:** Jenny H. Chang, Daphne Remulla, Chase Wehrle, Kimberly P. Woo, Fadi S. Dahdaleh, Daniel Joyce, Samer A. Naffouje

**Affiliations:** 1 Department of General Surgery, Cleveland Clinic Foundation, Cleveland, OH USA; 2 Department of Surgical Oncology, Edward-Elmhurst Health, Naperville, IL USA

**Keywords:** Merkel cell carcinoma, Neoadjuvant immunotherapy, National Cancer Database, Lymph node metastasis

## Abstract

**Background:**

Immunotherapy is emerging as a promising option for certain locally advanced and metastatic cutaneous malignancies. However, the role of neoadjuvant immunotherapy (NIO) in Merkel cell carcinoma (MCC) with clinically detected regional lymph node metastasis (CDRLNM) has not been fully elucidated.

**Methods:**

For this study, MCC patients with CDRLNM who underwent surgical excision were selected from the National Cancer Database (NCDB). Those who received NIO were propensity-matched with those who did not, and Kaplan-Meier analysis was used to compare overall survival (OS).

**Results:**

Of the 1809 selected patients, 356 (19.7%) received NIO followed by wide excision (*n* = 352, 98.9%) or amputation (*n* = 4, 1.1%). The rate of complete pathologic response for the primary tumor (ypT0) was 45.2%. Only 223 patents (63.4%) also underwent lymph node dissection (LND). The complete pathologic nodal response (ypN0) rate for these patients was 17.9%. A pathologic complete response of both the primary tumor and the nodal basin (ypT0 ypN0) was seen in 16 of the 223 patients who underwent both primary tumor surgery and LND. Subsequently, 151 pairs were matched between the NIO and no-NIO groups (including only patients with LND). Kaplan-Meier analysis demonstrated a significant OS improvement with NIO (median not reached vs. 35.0 ± 8.0 months; *p* = 0.025). The 5-year OS was 57% in the NIO group versus 44% in no-NIO group (*p* = 0.021).

**Conclusion:**

The study suggests that NIO in MCC with CDRLNM provides improved OS in addition to promising rates of primary complete response, which could change the profile of surgical resection. This supports ongoing clinical trials exploring the use of NIO in MCC.

Merkel cell carcinoma (MCC) is a clinically aggressive cutaneous malignancy of neuroendocrine origin. Despite the rarity of MCC relative to other cutaneous malignancies, the overall incidence has risen exponentially in the past few decades, with several population-based studies citing a 50–95% increase since the early 2000s.^1–3^ The risk factors for MCC include ultraviolet radiation exposure, chronic immunosuppression, and infection with Merkel cell polyomavirus (MCPyV).

At initial presentation, MCC is most often described as a painless, flesh-colored, rapidly growing nodule in a sun-exposed area.^4^ With its lack of distinguishing features and a high tendency for in-transit metastases,^5^ MCC is often diagnosed at an advanced stage, with more than 50% of patients presenting with nodal or distant metastases.^1,6^

The prognosis for patients with MCC is heavily dependent on the extent of nodal involvement. Historically, the 5-year overall survival (OS) decreases from 40.3% for stage IIIA disease with clinically occult nodes to 26.8% for stage IIIB disease with clinically detected nodes, and to 13.5% for stage IV disease with distant metastases due to limited systemic treatment options.^7,8^ As such, the National Comprehensive Cancer Network (NCCN) guidelines recommend sentinel lymph node biopsy (SLNB) whenever feasible, regardless of primary tumor size.^9^

Currently, the treatment paradigm for MCC with clinically detected regional lymph node metastases (CDRLNM) is surgical excision with adjuvant or definitive radiotherapy. However, recent landmark randomized controlled trials investigating melanoma have shown improved relapse-free survival (RFS) and event-free survival (EFS) using neoadjuvant immunotherapy (NIO) for stage III melanoma with clinically detected lymph node metastases.^10,11^

Although both MCC and melanoma are aggressive cutaneous malignancies with similar risk factors and evidence of tumor immunogenicity,^12,13^ data supporting the role of NIO in MCC with CDRLNM are very limited. To date, a single trial has been published, the CheckMate 358 trial, which demonstrated a 47.2% complete primary tumor response rate for patients with stages IIA to IV MCC who received neoadjuvant nivolumab.^14^ However, 3 of the 39 patients were unable to undergo surgery (1 patient due to a treatment-related adverse event related to NIO), and 46% of the patients reported any type of TRAE.

Currently, multiple other clinical trials are ongoing to evaluate different dosages and regimens of NIO.^15,16^

This study aimed to understand the trends of NIO use in MCC with CDRLNM on a national level using a cancer registry and to describe the impact of NIO on OS outcomes. We hypothesized that in line with findings from melanoma studies, offering neoadjuvant therapy to MCC patients who have CDRLNM with the tumor *in situ* provides better long-term OS than upfront excision and adjuvant therapy.

## Methods

This retrospective study used the National Cancer Database (NCDB), which captures hospital registry data collected in more than 1500 American College of Surgeons Commission on Cancer-accredited facilities.^17^ Of the non-melanoma cutaneous malignancies, MCC histology diagnosed between 2012 and 2019 was selected. The diagnosis of MCC was confirmed via International Classification of Diseases (ICD)-9 code 209.3 and ICD-10 code C44. The following series of inclusion and exclusion criteria were applied:

- Patients who underwent resection of the primary MCC were selected. This criterion included patients who had a wide excision or amputation.
- Patients who had reports on the associated nodal basin management were selected, including cases reported as “none.” Patients with a missing or unknown nodal management approach were removed from the dataset.
- Among patients who had nodal sampling, only those with an available report on the number of examined and positive nodes were selected.
- Patients with metastatic disease (stage IV) were excluded.
- Patients with clinically occult regional lymph node metastasis (i.e., stage I, II, or IIIA) were not considered for further matched Kaplan-Meier analysis. Only those with CDRLNM (stage IIIB) were selected at this step.

The CDRLNM population was divided into two subgroups based on the receipt of NIO. Baseline demographic and treatment characteristics were compared, and trends of NIO utilization were plotted for the duration of inclusion (2012–2019). A multivariable logistic regression was constructed to calculate a propensity score for the likelihood of receiving NIO based on age, sex, race, Charlson score, tumor location, clinical T and N stage, immunosuppression status, other neoadjuvant and adjuvant therapies, and surgical resection.

The patients with cN1 were matched 1:1 based on the propensity score following the nearest-neighbor method within a caliper width of 0.1 standard deviations. The matched subgroups (no NIO vs NIO) were compared to ensure adequate calibration. Mixed-effect modeling was used to compare continuous variables, and conditional logistic regression was used for categorical variables between the subgroups before and after the match. Kaplan-Meier was used to plot the OS curves for the matched groups. Median OS and 5-year OS were compared using the log-rank test and Fisher’s exact test. For this statistical analysis, SPSS v29.0 (SPSS, Armonk, NY, USA) was used, and an alpha lower than 0.05 was set as a threshold for statistical significance throughout the study.

## Results

**Fig. 1:**
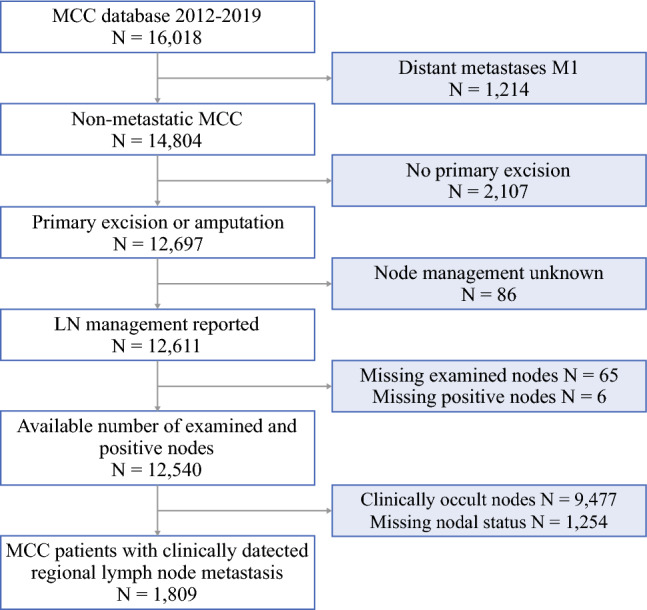
Flow diagram for the selection steps. LN, lymph nodes; MCC, Merkel cell carcinoma

The NCDB included 16,018 cases of cutaneous MCC diagnosed between 2012 and 2019. Application of the inclusion and exclusion criteria resulted in the selection of 1809 MCC patients with CDRLNM. Fig. 1 is a flow diagram summarizing the selection steps of the study’s patient population.

The population’s median age was 75 years, and about two thirds of the patients were males (*n* = 1269, 69.6%). Most MCCs were located in the head and neck area (*n* = 742, 41.0%). Of the 1809 MCC patients, 144 (8%) were reported to have profound immunosuppression for various reasons such as human immunodeficiency virus (HIV), transplantation, or liquid tumors, and 631 patients (34.9%) had tumors 2 cm in size or smaller (i.e., cT1). The majority of the cN+ diseases were attributed to palpable nodes or cN1 (*n* = 1638, 90.5%).

Of the 1809 patients with CDRLNM, 67 (3.7%) received neoadjuvant chemotherapy, and 356 (19.7%) received NIO. Only 24 patients (1.3%) received primary tumor radiation (pXRT), and 3 patients (0.2%) received nodal radiation (nXRT) in the neoadjuvant setting. All the patients subsequently received wide excision except 13 patients (0.7%) who received amputations.

For lymph node management, 1372 patients (75.8%) had lymph node dissection (LND), whereas 398 patients (22.0%) had no surgical intervention to the affected nodal basin, and 39 patients (2.2%) had sentinel lymph node biopsy (SLNB) after NIO. Table 1 summarizes the demographic and clinical characteristics of the selected patient population.

**Table 1:** Demographic and perioperative characteristics of MCC patients with CDRLNM

*n*		*n* (%) 1809
Age (years)	Mean ± SD	73.4 ± 11.2, 75
	Median (IQR)	75 (66–82)
Sex	Male	1259 (69.6)
	Female	550 (30.4)
Race	White	1682 (93.0)
	Black	25 (1.4)
	Other	102 (5.6)
Charlson score	0	1207 (66.7)
	1	385 (21.3)
	2	111 (6.1)
	3+	106 (5.9)
Location	Head & neck	742 (41.0)
	Trunk	246 (13.6)
	Upper extremity	439 (24.3)
	Lower extremity	336 (18.6)
	Overlapping/NOS	46 (2.5)
Clinical T stage	cT0	18 (1.0)
	cT1	631 (34.9)
	cT2	553 (30.6)
	cT3	135 (7.5)
	cT4	50 (2.8)
	cTx	422 (23.3)
Clinical N stage	Nodes (cN1)	1638 (90.5)
	In-transit (cN2)	95 (5.3)
	Both (cN3)	76 (4.2)
Immunosuppression	None	1047 (57.9)
	HIV	11 (0.6)
	Transplant	49 (2.7)
	Liquid malignancy	70 (3.9)
	Other	14 (0.8)
	Unknown	618 (34.2)
Neoadjuvant therapies	Chemotherapy	67 (3.7)
	Immunotherapy	356 (19.7)
	pXRT	24 (1.3)
	nXRT	3 (0.2)
Surgery	Wide excision	1796 (99.3)
	Amputation	13 (0.7)
Lymph node management	None	398 (22.0)
	SLNB	39 (2.2)
	LND	1372 (75.8)

MCC, Merkel cell carcinoma; CDRLNM, clinically detected regional lymph node metastasis; SD, standard deviation; IQR, interquartile range; NOS, not otherwise specified; cT0, *in situ* disease or no evidence of primary tumor;; cTx, unreported or missing data; HIV, human immunodeficiency virus; pXRT, primary radiation; nXRT, nodal radiation; SLNB, sentinel lymph node biopsy; LND, lymph node dissection;

Regarding the post-interventional outcomes for all the included patients with MCC and CDRLNM, the excision margins were positive in 338 of the patients (18.7%). A majority of the 1411 patients who underwent LND or SNLB (*n* = 1302, 92.3%) had positive nodal disease, and positive extranodal extension (ENE) was documented in 29.1% of the cases. The median number of examined nodes was 5 (interquartile range [IQR], 1–19), and the median number of positive nodes in the population was 1 (IQR, 0–3). One third of the patients (*n* = 580, 32.1%) proceeded to receive adjuvant systemic therapy (possible chemotherapy and/or immunotherapy), with 633 patients (35.0%) receiving pXRT and 156 patients (8.6%) receiving nXRT postoperatively. The median follow-up period was 27 months (IQR, 12–50 months). Table 2 presents the pathologic outcomes and postoperative treatment choices for the selected population.

**Table 2:** Pathologic and postoperative treatment details of MCC patients with clinically detected regional lymph node metastasis

*n*		*n* (%) 1809
Pathologic T stage	pT0	189 (10.4)
	pT1	641 (35.4)
	pT2	413 (22.8)
	pT3	155 (8.6)
	pT4	110 (6.1)
	pTx	301 (16.6)
Pathologic N status	Unknown	398 (22.0)
	Negative	109 (6.0)
	Positive	1302 (72.0)
Examined nodes	Mean ± SD	12.3 ± 16.0
	Median (IQR)	5 (1–19)
Positive nodes	Mean ± SD	3.0 ± 5.4
	Median (IQR)	1 (0–3)
Isolated tumor cells	No	1634 (90.3)
	Yes	175 (9.7)
Extranodal extension	No	1283 (70.9)
	Yes	526 (29.1)
Margins	Negative	1394 (77.1)
	Positive	338 (18.7)
	Unknown	77 (4.3)
Adjuvant therapy	Systemic	580 (32.1)
	pXRT	633 (35.0)
	nXRT	156 (8.6)
Follow-up	Mean ± SD	34.6 ± 27.2, 27
	Median (IQR)	27 (12–50)

**Fig. 2:**
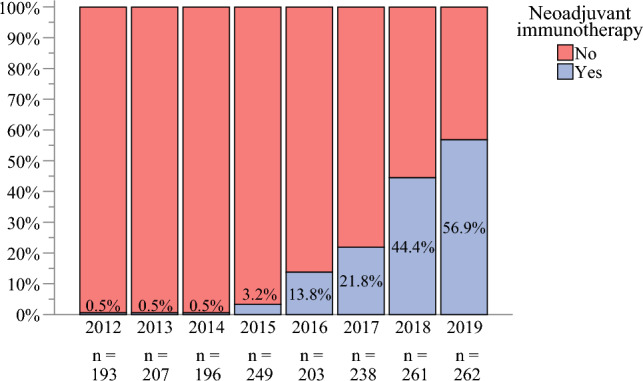
Trends of neoadjuvant immunotherapy utilization for MCC patients with CDRLNM during the selection period in the NCDB 2012–2019. MCC, Merkel cell carcinoma; CDRLNM, clinically detectable regional lymph node metastasis; NCDB, National Cancer Database

MCC, Merkel cell carcinoma; pT0, *in situ* disease or no evidence of primary tumor; pTx, unreported or missing data; SD, standard deviation IQR, interquartile range; pXRT, primary radiation; nXRT, nodal radiation;

We observed the chronological trends of offering NIO to MCC patients with CDRLNM and noted a significant increase from 0.5% in 2012 to 56.9% in 2019. These trends are demonstrated in Fig. 2. As shown in Table 3, 356 (19.7%) patients with MCC and CDRLNM (clinical nodal stage cN1) received NIO. Many of these patients were males (*n* = 255, 71.6%) and immunocompetent (*n* = 333, 93.5%). Compared with the patients who had MCC and CDRLNM without NIO, the patients with NIO had a lower Charlson comorbidity score (*p* = 0.02) and were more likely to be immunocompetent (93.5% vs 49.1%; *p* < 0.001), less likely to undergo LND (51.7% vs 81.8%; *p* < 0.001), and under other systemic adjuvant therapies (92.1% vs 17.3%; *p* < 0.001) (Table 3).

**Table 3:** Comparison of clinical and peri-operative characteristics of MCC patients with CDRLNM who did not receive neoadjuvant immunotherapy vs those who did before and after the propensity score-matching

	Unmatched dataset			Matched dataset 1:1		
	No NIO *n* (%)	NIO *n* (%)	*p* Value	No NIO *n* (%)	NIO *n* (%)	*p* Value
*n*	1453	356		151	151	
Age (years)	73.7 ± 11.1	72.2 ± 11.4	0.880	69.2 ± 9.8	70.2 ± 11.0	0.510
Sex			0.352			0.701
Male	1004 (69.1)	255 (71.6)		107 (70.9)	110 (72.8)	
Female	449 (30.9)	101 (28.4)		44 (29.1)	41 (27.2)	
Race			0.206			0.929
White	1358 (93.5)	324 (91.0)		137 (90.7)	138 (91.4)	
Black	20 (1.4)	5 (1.4)		4 (2.6)	3 (2.0)	
Other	75 (5.2)	27 (7.6)		10 (6.6)	10 (6.6)	
Charlson score			**0.023**^**a**^			0.402
0	953 (65.6)	254 (71.3)		94 (62.3)	99 (65.6)	
1	330 (22.7)	55 (15.4)		49 (32.5)	40 (26.5)	
2	85 (5.8)	26 (7.3)		6 (4.0)	10 (6.6)	
3+	85 (5.8)	21 (5.9)		2 (1.3)	2 (1.3)	
Location			0.086			0.617
Head & neck	617 (42.5)	125 (35.1)		47 (31.1)	52 (34.4)	
Trunk	197 (13.6)	49 (13.8)		29 (19.2)	20 (13.2)	
Upper extremity	344 (12.7)	95 (26.7)		44 (29.1)	48 (31.8)	
Lower extremity	262 (18.0)	74 (20.8)		28 (18.5)	26 (17.2)	
Overlapping/NOS	33 (2.3)	13 (3.7)		3 (2.0)	5 (3.3)	
Clinical T stage			0.124			0.608
cT0	13 (0.9)	5 (1.4)		2 (1.3)	2 (1.3)	
cT1	522 (35.9)	109 (30.6)		33 (21.9)	44 (29.1)	
cT2	450 (31.0)	103 (28.9)		48 (31.8)	46 (30.5)	
cT3	106 (7.3)	29 (8.1)		22 (14.6)	15 (9.9)	
cT4	41 (2.8)	9 (2.5)		4 (2.6)	2 (1.3)	
cTx	321 (22.1)	101 (28.4)		42 (27.8)	42 (27.8)	
Clinical N stage			**<0.001**^**a**^			1.000
cN1	1282 (88.2)	356 (100.0)		151 (100.0)	151 (100.0)	
cN2	95 (6.5)	0 (0.0)		0 (0.0)	0 (0.0)	
cN3	76 (5.2)	0 (0.0)		0 (0.0)	0 (0.0)	
Immunosuppression			**<0.001**^**a**^			0.101
No	714 (49.1)	333 (93.5)		129 (85.4)	138 (91.4)	
Yes	124 (8.5)	20 (5.6)		15 (9.9)	12 (7.9)	
Unknown	615 (42.3)	3 (0.8)		7 (4.6)	1 (0.7)	
Neoadjuvant therapies						
Chemotherapy	20 (1.4)	47 (13.2)	**<0.001**^**a**^	13 (8.6)	18 (11.9)	0.343
pXRT	20 (1.4)	4 (1.1)	0.709	1 (0.7)	1 (0.7)	1.000
nXRT	3 (0.2)	0 (0.0)	0.391	0 (0.0)	0 (0.0)	1.000
Surgery			0.313			1.000
Wide excision	1444 (99.4)	352 (98.9)		149 (98.7)	149 (98.7)	
Amputation	9 (0.6)	4 (1.1)		2 (1.3)	2 (1.3)	
Lymph node management			**<0.001**^**a**^			1.000
None	265 (18.2)	133 (37.4)		0 (0.0)	0 (0.0)	
SLNB	0 (0.0)	39 (11.0)		0 (0.0)	0 (0.0)	
LND	1188 (81.8)	184 (51.7)		151 (100.0)	151 (100.0)	
Adjuvant therapies						
Systemic	252 (17.3)	328 (92.1)	**<0.001**^**a**^	142 (94.0)	137 (90.7)	0.278
pXRT	503 (34.6)	130 (36.5)	0.501	60 (39.7)	60 (39.7)	1.000
nXRT	130 (8.9)	26 (7.3)	0.322	14 (9.3)	14 (9.3)	1.000

MCC, Merkel cell carcinoma; CDRLNM, clinically detected regional lymph node metastasis; NIO, neoadjuvant immunotherapy; NOS, not otherwise specified; cT0, *in situ* disease or no evidence of primary tumor; cTx, unreported or missing data; pXRT, primary radiation; nXRT, nodal radiation; SLNB, sentinel lymph node biopsy; LND, lymph node dissection

^a^Statistically significant

When the pathologic outcomes for the patients who received NIO (*n* = 356) were examined, the primary tumor (ypT0) showed a complete response rate of 45.2%, a partial response (ypT downstaging) rate of 17.4%, and a no response (ypT = cT) rate of 37.4%. Among the NIO patients who had LND (*n* = 223), negative nodal status was documented on pathology for 17.9% (*n* = 40). The remaining 82.1% (*n* = 183) had persistent positive nodal status, 30.9% of whom had additional extranodal extension.

**Fig. 3:**
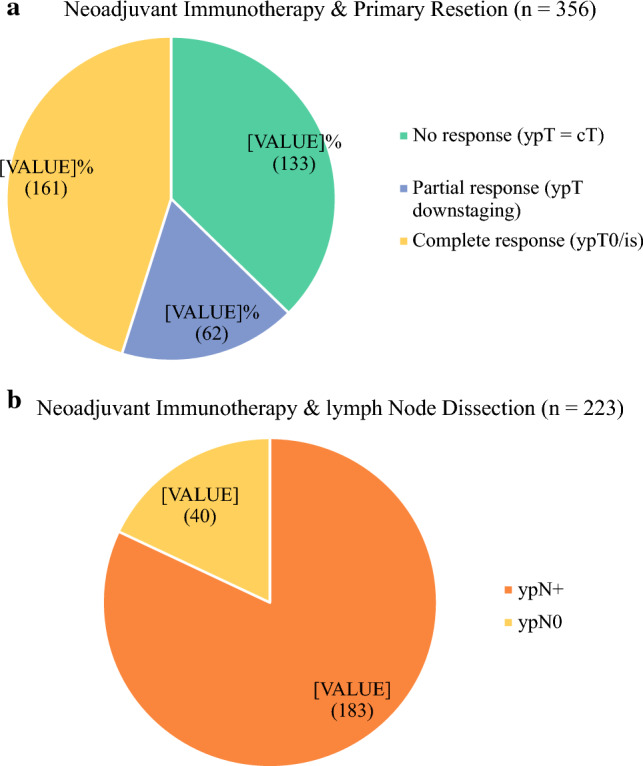
Pathologic outcomes after neoadjuvant immunotherapy for MCC patients with clinically detected regional lymph node metastasis. **a** Neoadjuvant immunotherapy and primary resection (*n* = 356). **b** Neoadjuvant immunotherapy and lymph node dissection (*n* = 223). MCC, Merkel cell carcinoma; CR, complete response; NIO, neoadjuvant immunotherapy; NR, no response; PR, partial response

A complete pathologic response (i.e., pCR or ypT0 ypN0) was observed in 7.2% (*n* = 16). Figure 3 demonstrates the pathologic outcomes for the NIO patients who had resection of the primary tumor and those who had LND.

The selected population then was divided into two subgroups based on the receipt of NIO (*n* = 356) versus no NIO (*n* = 1453). The NIO patients were more likely to have a Charlson score of 0, to be immunocompetent, and to have received neoadjuvant chemotherapy. All the NIO patients had cN1 (i.e., palpable nodes), whereas none had cN2 (in-transit disease) or cN3 (both).

A propensity score was calculated based on a multivariable logistic regression, as described in the Methods section, and 151 pairs of patients were matched between the groups. Comparative analysis of the matched dataset confirmed resolution of all baseline differences, indicating adequate calibration. Notably, the match was applied under the condition of including only patients who had LND in both groups to avoid the confounding impact of not treating the lymph node basin on OS. Table 3 shows the comparison between the no-NIO and NIO subgroups in the unmatched and matched datasets.

**Fig. 4:**
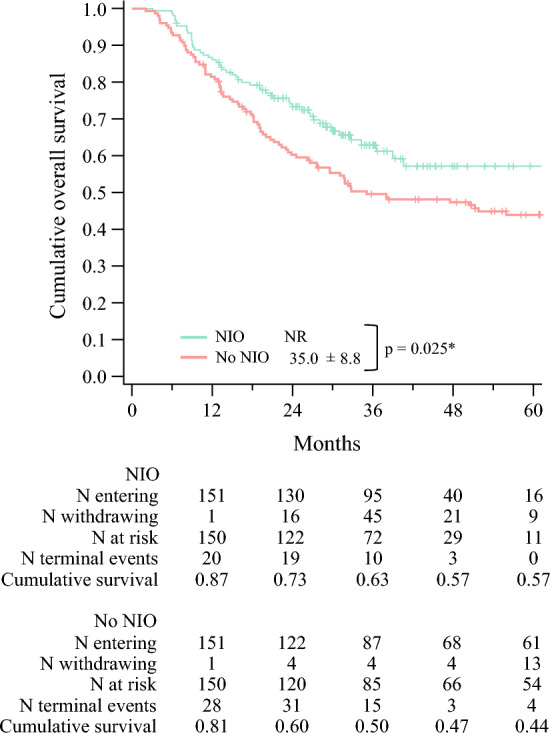
Kaplan-Meier analysis of overall survival in the matched dataset of MCC patients with CDRLNM who received neoadjuvant immunotherapy versus their matched peers who did not. MCC, Merkel cell carcinoma; CDRLNM, clinically detected regional lymph node metastasis; NIO, neoadjuvant immunotherapy; NR, not reached *Statistically significant

After matching, we performed a Kaplan-Meier analysis to compare OS between the matched groups. The NIO group had significantly better OS than the no-NIO group (median OS not reached vs 35.0 ± 8.0 months, respectively; *p* = 0.025). The absolute 5-year OS benefit in the NIO group was measured at 13% (57% vs 44%; *p* = 0.021). Figure 4 demonstrates the Kaplan Meier survival analysis of the matched groups of NIO vs no NIO in MCC patients with CDRLNM.

## Discussion

This large database study demonstrated a significant improvement in OS with the application of neoadjuvant immunotherapy for clinically node-positive MCC, even after adjustment for changes in oncologic presentation that may have indicated the need for this therapy. In 151 matched pairs of MCC patients with CDRLNM who underwent nodal dissection, our Kaplan-Meier analysis demonstrated an absolute survival benefit of 13% with NIO.

The benefits of NIO include potential downstaging and pathologic complete response rates that could improve the ability of patients to undergo surgical resection successfully, as well as decreased morbidity and improved tissue preservation. Beyond these traditional advantages of any neoadjuvant regimen, NIO is hypothesized in other malignancies to promote a more robust host immune response before the macroscopic removal of the tumor by activating both the priming phase of immunity within tumor tissues and the effector phase within the tumor microenvironment.^18,19^ This has been demonstrated in studies of multiple different carcinomas.

Due to the rarity of MCC relative to other cutaneous malignancies, suitable clinical trials are lacking, and data on the management of MCC is often inferred from melanoma studies, including the landmark OpACIN and SWOG S1801 trials. The OpACIN phase Ib trial showed that neoadjuvant treatment with nivolumab plus ipilimumab is feasible and induces a stronger and broader T cell response,^10^ which was followed by OpACIN-neo and PRADO trials.^20^ Consistent with this hypothesis of a larger anti-tumor immune response and longer immunologic memory with NIO, the SWOG S1801 trial examined the use of pembrolizumab, an anti-PD-1 monoclonal antibody, and demonstrated improved event-free survival for patients who had stages IIIB to IV resectable melanoma treated with neoadjuvant and adjuvant pembrolizumab versus adjuvant pembrolizumab alone.^11^

Inspired by these studies, we considered that NIO may represent a promising treatment paradigm for MCC patients with CDRLNM, as demonstrated by the gradual increase in practice pattern use of NIO in our study from 0.5% in 2014 to 56.9% in 2019. Because MCC is a particularly profoundly immunogenic tumor, the role of immunotherapy is strongly suggested in clinical studies, particularly in the adjuvant or metastatic setting of MCC, including the KEYNOTE-017 trial,^21^ the JAVELIN Merkel 200 trials,^22,23^ and the ADMEC-O trial.^24^ The KEYNOTE-017 study was a 2016 phase 2 trial that demonstrated a 56% complete or partial response rate during a median of 33 weeks of follow-up evaluation for patients who received at least one dose of pembrolizumab as first-line treatment for advanced MCC, with a 3-year overall survival of 90% for responders.^21,25^ The JAVELIN Merkel 200 trials, also published in 2016, demonstrated a 33% response rate for patients with metastatic MCC that progressed on chemotherapy treated with avelumab.^23^ The ADMEC-O trial, published in 2023, evaluated adjuvant nivolumab with an 84% disease-free survival rate for the patients in the immunotherapy group versus 73% for the control group during 24 months of follow-up evaluation.^24^

However, the Checkmate 358 trial is the only published trial to date that evaluated nivolumab in the neoadjuvant setting for MCC.^14^ The patients with stages IIA to IV resectable disease received two doses of nivolumab followed by definitive surgery. Of the 36 patients who underwent surgery, 17 (47%) achieved a pathologic complete response of the primary tumor with no relapse during the 20-month study period.^14^ Significant in our study and consistent with the Checkmate 358 trial, 45% of our patients had a complete primary tumor response after NIO, with improved overall survival. Although the current practice remains consistent with excision of the primary tumor and dissection of the nodal basin for those with CDRLNM despite a complete clinical response after NIO,^9^ there may be a future role for a watch and wait strategy, particularly for those with cancers in areas wherein surgical resection, radiation, or both may be challenging.

Yet, although not included in the NCDB, understanding the adverse events of immunotherapy also is an important consideration when its use for MCC is discussed. The KEYNOTE-017 trial noted that 98% of patients experienced treatment-related adverse events, including one treatment-related death.^25^ In the ADMEC-O trial, treatment-related adverse events occurred for 74% of the patients in the nivolumab group.^24^ Furthermore, in the Checkmate 358 trial, three patients (7.7%) receiving NIO ultimately did not undergo surgery despite enrolling with resectable disease due to tumor progression or adverse events.^14^

It should be noted that the patients in our study had higher 5-year OS rates in both the NIO (57%) and no NIO (44%) groups than in other studies of MCC patients with CDRLNM, such as the widely cited 5-year OS rates of 26% to 42% for clinically detected regional lymph node metastasis from the American Joint Committee on Cancer (AJCC).^7^ However, in addition to stage, significant survival differences persist when patients are further stratified by age and site of MCC.^1^ This may be attributable to poor historical survival due to limited systemic treatment options and the selection bias in our study. Our selection of patients who underwent surgical excision may have been inherently skewed toward a subset of patients who may have had favorable prognostic factors or better overall health. We controlled for this by using the surrogate Charlson score in our propensity score-matched analysis, with both groups having a higher survival rate.

This study had several other limitations inherent to the use of national registries. There are two different etiologies of MCC^26^ that are not reported in the NCDB. Up to 80% of MCC tumors are positive for clonal integration and expression of MCPyV viral proteins^26,27^ which may affect the response of MCC to immunotherapy.

Other predictive markers such as PD-L1 or tumor mutational burden, which are not available in the NCDB, should be included in the decision to initiate immunotherapy in a multidisciplinary setting. Currently, multiple ongoing clinical trials are exploring this,^16^ although in the KEYNOTE-017 trial, tumor viral or PD-L1 status was not associated with improved overall survival.^25^ This is in contrast to a study demonstrating that patients with positive MCPyV status were more likely to express PD-L1 and have improved overall survival.^28^

A strong link exists between T cell immunity and progression of MCC because patients who are T cell immunosuppressed with MCC have more than double the risk of disease-specific mortality.^29^ However, nearly all patients who underwent neoadjuvant immunotherapy in this study were immunocompetent. This likely was due to limited knowledge on the efficacy and safety of immunotherapy for immunosuppressed patients, who typically are excluded from clinical trials. In one systematic review of solid organ transplant recipients with metastatic cutaneous cancers undergoing immunotherapy, 37% of the patients experienced graft reaction.^30^

Details regarding the duration and type of NIO used as well as treatment-adverse effects also are not reported to the NCDB. Although a single-agent immunotherapy is considered in advanced or metastatic MCC, to date, the optimal therapy is not defined, with several available checkpoint inhibitors available to use for MCC. These include several anti-PD-L1 and anti-PD-1 monoclonal antibodies such as avelumab, pembrolizumab, retifanlimab, and nivolumab. Additionally, it should be noted that 92% of the patients in the unmatched NIO group had other adjuvant systemic therapies, and the role of NIO in combination of adjuvant systemic therapy was not elucidated.

Despite the shortcomings of missing data points or misreporting in the NCDB data, for rare diseases such as MCC, institutional studies often report small sample sizes that preclude the drawing of definitive conclusions. Therefore, large registry studies complement the diversity of research analyzing such rare diseases by accruing patients on a national level and applying large-scale analytics. We aimed to compensate for these deficiencies by performing a strict propensity score match to adjust for all reported confounders. Studies on NIO use in MCC (e.g., neoadjuvant cemiplimab, ClinicalTrials.gov ID NCT04975152; and neoadjuvant nivolumab and relatlimab, ClinicalTrials.gov ID NCT06151236) together with predictive markers such as MCPyV status, PD-L1, or tumor mutational status to determine MCC immunotherapy efficacy are ongoing.^15,16^ Also of future interest is an optimal regimen for NIO and the prognostic and practice-changing implications for patients who may have a complete pathologic response to NIO.

## Conclusion

Our study showed that use of NIO for MCC patients with CDRLNM has been steadily increasing on a national level in the past few years, with promising clinical responses. Furthermore, our analysis showed that NIO provides OS benefit for stage IIIB MCC patients and should be considered as a new treatment paradigm for eligible patients supported by ongoing clinical trials.
